# Metabolic Fingerprints of Altered Brain Growth, Osmoregulation and Neurotransmission in a Rett Syndrome Model

**DOI:** 10.1371/journal.pone.0000157

**Published:** 2007-01-17

**Authors:** Angèle Viola, Véronique Saywell, Laurent Villard, Patrick J. Cozzone, Norbert W. Lutz

**Affiliations:** 1 Centre de Résonance Magnétique Biologique et Médicale (CRMBM), UMR Centre National de la Recherche Scientifique (CNRS) 6612, Université de la Méditerranée, Faculté de Médecine, Marseille, France; 2 Institut National de la Santé et de la Recherche Médicale (INSERM) U491, Faculté de Médecine de la Timone, Marseille, France; 3 Université de la Méditerranée, Faculté de Médecine de la Timone, Marseille, France; Laboratory of Neurogenetics, National Institutes of Health, United States of America

## Abstract

**Background:**

Rett syndrome (RS) is the leading cause of profound mental retardation of genetic origin in girls. Since RS is mostly caused by mutations in the *MECP2* gene, transgenic animal models such as the *Mecp2*-deleted (“*Mecp2*-null”) mouse have been employed to study neurological symptoms and brain function. However, an interdisciplinary approach drawing from chemistry, biology and neuroscience is needed to elucidate the mechanistic links between the genotype and phenotype of this genetic disorder.

**Methodology/Principal Findings:**

We performed, for the first time, a metabolomic study of brain extracts from *Mecp2*-null mice by using high-resolution magnetic resonance spectroscopy. A large number of individual water-soluble metabolites and phospholipids were quantified without prior selection for specific metabolic pathways. Results were interpreted in terms of *Mecp2* gene deletion, brain cell function and brain morphology. This approach provided a “metabolic window” to brain characteristics in *Mecp2*-null mice (n = 4), revealing (i) the first metabolic evidence of astrocyte involvement in RS (decreased levels of the astrocyte marker, *myo*-inositol, vs. wild-type mice; p = 0.034); (ii) reduced choline phospholipid turnover in *Mecp2*-null vs. wild-type mice, implying a diminished potential of cells to grow, paralleled by globally reduced brain size and perturbed osmoregulation; (iii) alterations of the platelet activating factor (PAF) cycle in *Mecp2*-null mouse brains, where PAF is a bioactive lipid acting on neuronal growth, glutamate exocytosis and other processes; and (iv) changes in glutamine/glutamate ratios (p = 0.034) in *Mecp2*-null mouse brains potentially indicating altered neurotransmitter recycling.

**Conclusions/Significance:**

This study establishes, for the first time, detailed metabolic fingerprints of perturbed brain growth, osmoregulation and neurotransmission in a mouse model of Rett syndrome. Combined with morphological and neurological findings, these results are crucial elements in providing mechanistic links between genotype and phenotype of Rett syndrome. Ultimately, this information can be used to identify novel molecular targets for pharmacological RS treatment.

## Introduction

Rett syndrome (RS) is an X-linked neurological disorder that almost exclusively affects the female population. RS is the leading cause of profound mental retardation of genetic origin in girls. First described in the 1960s by A. Rett [1], this disorder has been recognized as a neurological syndrome in the 1980s [2], and its genetic basis has been discovered very recently [3]. In up to 96% of all classic cases, RS is caused by mutations or deletions in *MECP2*, a gene encoding for the MECP2 (methyl cytosine phosphate dinucleotide guanine binding) protein which acts as a transcriptional repressor critical for normal neuronal function [4], [5].

Like other genetic disorders, RS is characterized by abnormal expression of a gene, resulting in a clinical phenotype. Consequently, research on genotype-phenotype correlation, i.e. the association between the presence of a certain mutation (genotype) and the resulting traits (phenotype), is crucial for establishing the basic link between altered gene expression and the disorder. Beyond genotype-phenotype correlation studies, the elucidation of actual mechanisms leading from particular genetic changes to specific symptoms is a rather challenging task. Multiple levels are involved including gene transcription, protein expression and posttranslational protein modification, all resulting in changes of the complex metabolic network that determines how cells perform specific tasks. For this reason, an interdisciplinary approach drawing from biochemistry, molecular biology, neuroscience and medicine is needed, with particular emphasis on the comprehensive analysis of cell metabolism.

While the spectrum of postnatal neurological disorders caused by *MECP2* mutations is rather broad [5], a number of behavioral, neuroanatomical and neurochemical features are consistently associated with classic RS. Affected girls appear to be normal until age 6–18 mo [4], [5]. Their neurological development is then arrested and begins regressing [6]. Symptoms include decelerating head growth, resulting in autistic features, loss of skills such as speech and purposeful hand use, irregular breathing patterns, and in many cases seizures. Morphometric and volumetric studies of subjects with RS showed microcephaly and size reductions of certain brain regions [7]–[9]. In fact, the most recent ‘clinically applicable diagnostic criteria in RS’ include ‘a deceleration of head growth of two standard deviations’ as one of the consensus criteria [10], [11]. Cerebellar atrophy has been detected in adult RS patients [12]. Histological investigations have revealed a reduction in neuron size and in dendritic arborization [7]. Several groups have conducted genotype-phenotype correlation studies to determine whether different types of mutations in *MECP2* can account for the variability of clinical features in RS patients. However, these studies have yielded inconsistent results [4]. To enable more systematic and better-controlled investigations, transgenic mouse models have been engineered that are characterized by mutated *Mecp2* [5], [13]–[16]. Among these, the *Mecp2*-null mouse model is widely accepted [13]–[16]. All these models have been employed to study neurological symptoms, respiration, synaptic transmission, neurotransmitter receptors, neuronal architecture and morphology, but also the involvement of astrocytes [4], [5], [17]–[19].

In a previous study, we have used the *Mecp2*-null mouse model to determine the cerebral endophenotype associated with the lack of the Mecp2 protein in brain by using in vivo magnetic resonance imaging (MRI) and spectroscopy (MRS) [20]. Volumetric analysis of MRI images have revealed important neuroanatomical anomalies such as a size reduction of the whole brain and of structures involved in cognitive and motor functions (cerebellum and motor cortex) in *Mecp2*-/y vs. wild-type mice. These observations reflect in part changes detected previously in RS patients. Several groups have used localized ^1^H MRS methods to measure variations of *N*-acetylaspartate (NAA), choline, *myo*-inositol (*myo*-Ins) and glutamate/glutamine levels in different brain regions of RS patients [21]–[23]. Our in vivo MRS studies have yielded neurochemical differences between wild-type and *Mecp2*-/y mice, although the number of metabolites that can be quantitated in vivo is rather limited. We now present a detailed metabolomic study based on high-resolution ^1^H and ^31^P MRS of brain *extracts*.

The principal purpose of this pilot study is to provide a comprehensive analysis of the brain metabolic phenotype associated with *Mecp2* deletion [24], and to relate these results to morphological and neurological characteristics. Like other ‘omics’ (e.g. genomics, proteomics), metabolomics has the benefit of simultaneously providing a broad overview of molecular processes without prior selection for specific pathways [25]. Notably, we report the first phospholipidomic analysis of brain tissue characterized by a mutation of *MeCP2* in humans or animals. This approach permits the detection of characteristic “fingerprints” of metabolic processes. These in turn provide a sound basis for further mechanistic studies by establishing well-founded hypotheses. Ultimately, the detection of metabolic processes related to RS features such as cerebral atrophy and neuronal dysfunction should enable the identification of molecular targets for more efficient pharmacological treatment of the disorder.

## Results

Significant differences between Mecp2-deficient mice and controls were detected for a number of metabolites. While the presentation of results given below is organized according to biochemical categories, it should be kept in mind that most metabolites are involved in *multiple* metabolic pathways and processes. Thus, the context provided for individual metabolites should not be considered exhaustive.

### Mecp2 deletion alters the brain profile of choline-containing phospholipids and cardiolipin

**Figure 1:**
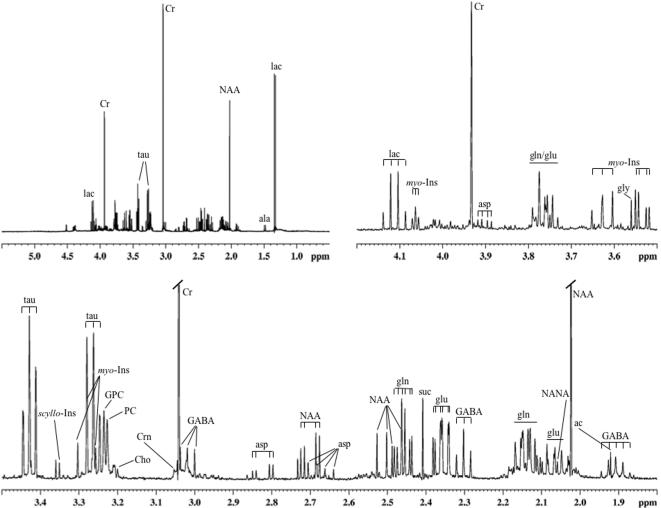
Typical high-resolution ^1^H NMR spectrum of the brain extract from a Mecp2-deficient mouse (water-soluble metabolites). The entire spectral region of interest (upper left spectrum) as well as magnified subregions are shown. Creatinine (Crn) was not quantified since the weak singlet was not sufficiently separated from other peaks forming a broad hump between 3.01 and 3.06 ppm. Abbreviations: ac, acetate; ala, alanine; asp, aspartate; Cho, choline; Cr, creatine; GABA, γ-aminobutyrate; gln, glutamine; glu, glutamate; gly, glycine; GPC, glycerophosphocholine; lac, lactate; *myo*-Ins, myo-inositol; NAA, *N*-acetylaspartate; NANA, *N*-acetylneuraminate; PC, phosphocholine; *scyllo*-Ins, *scyllo*-inositol; suc, succinate; tau, taurine.

Our previous in vivo ^1^H MRS study revealed altered relative levels of total choline (tCho) in Mecp2-deficient mice vs. controls [20], where tCho represents several metabolites predominantly involved in choline phospholipid metabolism (choline, Cho; phosphocholine, PC; glycerophosphocholine, GPC), plus substantial contributions from phosphoethanolamine and taurine [25]. The phosphodiester, glycerophosphocholine, is a phospholipid (PL) degradation product while choline and the phosphomonoester, phosphocholine, are both phospholipid anabolites and catabolites. Since individual choline compounds and phospholipid classes cannot be quantitated by in vivo MRS, we determined their (absolute) tissue levels by ^1^H MRS of whole-brain extracts (Fig. 1). Each of the three choline species mentioned above showed a trend towards decreased levels in Mecp2-deficient mice vs. controls (Table 1); the difference was close to statistical significance for choline (ca. −40%; p = 0.077), but not for the phosphorylated choline species. For the sum of these three choline compounds, a slight decrease was observed (not significant), in agreement with our previous in vivo results obtained at a short echo time [20]. Interestingly, the PC/GPC ratio was clearly increased by 30% (p = 0.034), mostly due to a GPC decrease (Table 1).

**Table 1:**
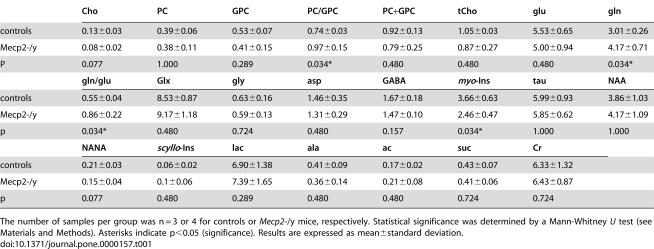
Metabolite levels in Mecp2-/y and control mouse brain (µmol/g wet weight, and molar ratios for selected metabolites)

	Cho	PC	GPC	PC/GPC	PC+GPC	tCho	glu	gln
controls	0.13±0.03	0.39±0.06	0.53±0.07	0.74±0.03	0.92±0.13	1.05±0.03	5.53±0.65	3.01±0.26
Mecp2-/y	0.08±0.02	0.38±0.11	0.41±0.15	0.97±0.15	0.79±0.25	0.87±0.27	5.00±0.94	4.17±0.71
P	0.077	1.000	0.289	0.034*	0.480	0.480	0.480	0.034*
	**gln/glu**	**Glx**	**gly**	**asp**	**GABA**	***myo*-Ins**	**tau**	**NAA**
controls	0.55±0.04	8.53±0.87	0.63±0.16	1.46±0.35	1.67±0.18	3.66±0.63	5.99±0.93	3.86±1.03
Mecp2-/y	0.86±0.22	9.17±1.18	0.59±0.13	1.31±0.29	1.47±0.10	2.46±0.47	5.85±0.62	4.17±1.09
p	0.034*	0.480	0.724	0.480	0.157	0.034*	1.000	1.000
	**NANA**	***scyllo*** **-Ins**	**lac**	**ala**	**ac**	**suc**	**Cr**	
controls	0.21±0.03	0.06±0.02	6.90±1.38	0.41±0.09	0.17±0.02	0.43±0.07	6.33±1.32	
Mecp2-/y	0.15±0.04	0.1±0.06	7.39±1.65	0.36±0.14	0.21±0.08	0.41±0.06	6.43±0.87	
p	0.077	0.480	0.289	0.480	0.480	0.724	0.724	

The number of samples per group was n = 3 or 4 for controls or *Mecp2*-/y mice, respectively. Statistical significance was determined by a Mann-Whitney *U* test (see Materials and Methods). Asterisks indicate p<0.05 (significance). Results are expressed as mean±standard deviation.

**Figure 2:**
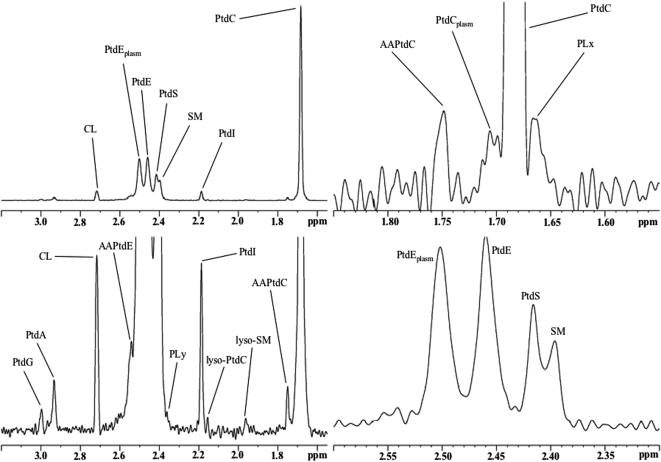
Typical high-resolution ^31^P NMR spectrum of the brain extract from a Mecp2-deficient mouse (phospholipids). The entire PL region (upper left spectrum) as well as magnified subregions are shown. Processing parameters were varied to highlight important spectral details. Abbreviations: AAPtdC, alkyl-acyl-phosphatidylcholine; AAPtdE, alkyl-acyl-phosphatidylethanolamine; CL, cardiolipin; PLx,y, unassigned phospholipids; PtdI, phosphatidylinositol; PtdA, phosphatidic acid; PtdC, (diacyl)phosphatidylcholine; PtdC_plasm_, choline plasmalogen; PtdE, (diacyl)phosphatidylethanolamine; PtdE_plasm_, ethanolamine plasmalogen; PtdG, phosphatidylglycerol; PtdS, phosphatidylserine; SM, sphingomyelin.

^31^P MRS was able to distinguish 16 phospholipid classes and subclasses (Fig. 2), fourteen of which were successfully assigned. The analysis of PL profiles by ^31^P MRS showed significant differences in PL levels between Mecp2-deficient mice and controls solely for choline-containing PL (diacyl-phosphatidylcholine, PtdC; alkyl-acyl-phosphatidylcholine, AAPtdC; choline plasmalogen, PtdC_plasm_; lyso- phosphatidylcholine, lyso-PtdC) (Table 2; see also Table S1 for cardiolipin, CL).

**Table 2:**
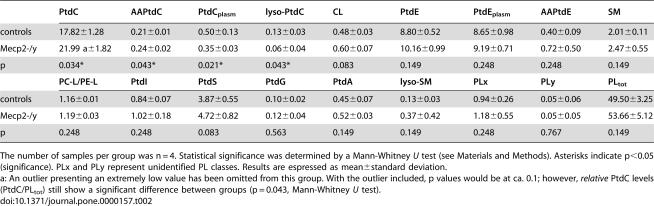
Metabolite levels in Mecp2-/y and control mouse brain (µmol/g wet weight, and molar ratios for selected metabolites)

	PtdC	AAPtdC	PtdC_plasm_	lyso-PtdC	CL	PtdE	PtdE_plasm_	AAPtdE	SM
controls	17.82±1.28	0.21±0.01	0.50±0.13	0.13±0.03	0.48±0.03	8.80±0.52	8.65±0.98	0.40±0.09	2.01±0.11
Mecp2-/y	21.99 a±1.82	0.24±0.02	0.35±0.03	0.06±0.04	0.60±0.07	10.16±0.99	9.19±0.71	0.72±0.50	2.47±0.55
p	0.034*	0.043*	0.021*	0.043*	0.083	0.149	0.248	0.248	0.149
	**PC-L/PE-L**	**PtdI**	**PtdS**	**PtdG**	**PtdA**	**lyso-SM**	**PLx**	**PLy**	**PL_tot_**
controls	1.16±0.01	0.84±0.07	3.87±0.55	0.10±0.02	0.45±0.07	0.13±0.03	0.94±0.26	0.05±0.06	49.50±3.25
Mecp2-/y	1.19±0.03	1.02±0.18	4.72±0.82	0.12±0.04	0.52±0.03	0.37±0.42	1.18±0.55	0.05±0.05	53.66±5.12
p	0.248	0.248	0.083	0.563	0.149	0.149	0.248	0.767	0.149

The number of samples per group was n = 4. Statistical significance was determined by a Mann-Whitney *U* test (see Materials and Methods). Asterisks indicate p<0.05 (significance). PLx and PLy represent unidentified PL classes. Results are espressed as mean±standard deviation.

a: An outlier presenting an extremely low value has been omitted from this group. With the outlier included, p values would be at ca. 0.1; however, *relative* PtdC levels (PtdC/PL_tot_) still show a significant difference between groups (p = 0.043, Mann-Whitney *U* test).

All other phospholipid classes and subclasses determined were present at very similar levels in the brains of both animal groups (diacyl-phosphatidylethanolamine, PtdE; alkyl-acyl-phosphatidylethanolamine, AAPtdE; ethanolamine plasmalogen, PtdE_plasm_; sphingomyelin, SM; lyso-sphingomyelin, lyso-SM; phosphatidylserine, PtdS; phosphatidylinositol, PI; phosphatidic acid, PtdA; phosphatidylglycerol, PtdG). Choline plasmalogen (PtdC_plasm_) was decreased in Mecp2-deficient mouse brain (Table 2); however, PtdC_plasm_ values may be less accurate than the other PL values since the PtdC_plasm_ peak areas measured may be influenced by the presence of the large PtdC signal directly upfield from PtdC_plasm_. PtdS and PtdE showed a trend towards increased values (not significant). PL_tot_ (total phospholipids) levels did not vary between groups, nor did the molar ratio between choline and ethanolamine-containing phospholipids, PC-L/PE-L (Table 2). As a matter of fact, molar fractions of phospholipids are a direct measure of the balance of phospholipid composition that affects the fluidity and function of membranes (Farooqui and Horrocks 2001).

### Cerebral profiles of neurotransmitters in Mecp2-null mice

Choline, introduced above as a phospholipid metabolite, is also involved in neurotransmitter metabolism as it is an anabolite and catabolite of acetylcholine. Other neurotransmitters and their metabolites are closely linked to glucose (glc) metabolism. Glutamate (glu), generally acknowledged to be the most important transmitter for normal brain function [26], can be produced from the citric acid cycle following pyruvate formation from glucose. We did not detect a significant difference in glutamate between Mecp2-deficient and control mice based on ^1^H spectra of brain extracts. However, the presynaptic precursor of glutamate, glutamine (gln), was significantly increased in Mecp2-deficient mice by almost 40%. The glutamine/glutamate ratio also was significantly increased in Mecp2-deficient mice (Table 1). Aspartate (asp) is a neurotransmitter and a glutamate metabolite, and, like glutamate, does not differ between Mecp2-deficient and control mice. Similarly, the inhibitory neurotransmitter, γ-aminobutyrate (GABA), is derived from glutamate and only shows an insignificant decrease in Mecp2-deficient vs. control mice. Glycine (gly), another neurotransmitter [27], shows almost identical concentrations in both groups.

### Mecp2 deletion alters cerebral osmolyte profiles

NAA is considered a neuronal marker and has also been proposed to act as an osmolyte [28]. NAA is involved in asp metabolism and lipid synthesis, and was found in large quantities in mouse brain (ca. 4 µmol/g). However, no differences were detected between the two groups (Table 1). Besides GPC and glutamine mentioned above, we quantitated two additional compounds known as osmolytes, taurine (tau) and *myo*-Ins. This polyol that is also important in phospholipid (phosphatidylinositol) metabolism, was decreased by one third in Mecp2-deficient mice (p = 0.034 for both significance tests, Table 1). In contrast, there was no difference in taurine levels between the two groups studied. Neither the NAA and ketone body-derived metabolite, acetate (ac), nor the glycolysis products, lactate (lac) and alanine (ala), nor citric acid cycle-derived succinate (suc) were different for the two groups analyzed. In a previous paper, we raised the possibility that the NAA signal measured by in vivo MRS may overlap with an unresolved signal from *N*-acetylaspartylglutamate (NAAG) [20]. The latter should be readily detectable as a separate resonance in brain extract spectra. Nevertheless, we did not find any peak next to NAA that would be intense enough to explain the additional signal measured in vivo (max. 5–6% of NAA). Therefore, the extra peak observed in vivo is, in all likelihood, not due to NAAG. Another *N*-acetyl compound, *N*-acetylneuraminate (NANA) or sialic acid, is moderately decreased in *Mecp2*-/y mice (near significance, p = 0.077, Table 1). NANA is synthesized from glucose and is involved in the synthesis of gangliosides that are implicated in signal transduction in eukaryotic cells [29]. Knockout mice lacking the major gangliosides of brain are highly susceptible to induced seizures [30]; however, little is known about a specific role of NANA in Rett syndrome.

## Discussion

A metabolic pattern that is based on a number of metabolites implicated in a common pathway can be considered a “fingerprint” of the metabolic process involved. Our metabolomic analysis revealed that the concentrations of several brain metabolites involved in different pathways varied significantly between *Mecp2*-null mice and controls. The biochemical processes include primarily membrane lipid metabolism, osmoregulation and glutamatergic neurotransmission. These will be interpreted in the context of morphological, neurological and functional differences between normal brains and brains characterized by *Mecp2* deletion.

### How are membrane phospholipids and brain (cell) growth linked in Mecp2-/y mice?

To our knowledge, the present study contains the first detailed quantitative analysis of membrane phospholipid classes and subclasses (phospholipidomics) in brain tissue with a mutated *Mecp2* or *MECP2* gene. PL metabolism plays an essential role in cell growth since PL form the matrix of cell membranes, and membrane synthesis is indispensible for cell growth. In fact, brain growth defects resulting in decreased head size and reduced neuronal cell size are one of the hallmarks of RS [7]. Global reduction in brain size and several of the morphological anomalies detected in RS patients have also been observed in the Mecp2-deficient mice used in this study [20]. Moreover, knockdown of Mecp2 levels in dissociated embryonic non-neuronal cells from mice has been shown to reduce their proliferation [19]. These effects point to growth perturbation in Mecp2-deficient brain tissue, and should therefore be reflected by parameters characterizing the biosynthesis and degradation of cellular membranes. Nonetheless, studies of cerebral membrane lipids in RS have been scarce, and have not included quantitation of individual phospholipid classes [31]–[33].

The individual steps of PtdC synthesis and degradation are well known (Kennedy pathway, Figure S1), and the role of PtdC turnover in membrane production and cell growth is well established. Therefore, the PtdC metabolite pattern observed in Mecp2-deficient mouse brain can be interpreted in terms of membrane PtdC turnover and cell growth, as outlined in the legend to Figure S1. The metabolic fingerprint of a reduced PtdC turnover rate indicates that the brain cells of Mecp2-deficient mice possess a reduced potential to boost PtdC production when cells are in a growing state [34], [35]. This may limit the ability of cells to grow, a mechanism potentially underlying brain growth arrest in Mecp2-deficient mice (see scheme below).


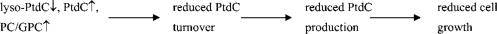


Autopsy of both RS patients and *Mecp2* mutant mice has revealed that the brain size is reduced without reduction of the number of neurons [36]. However, the size and dendritic arborization of neurons are reduced. On the other hand, there are up to ten times less neurons than glial cells (mainly astrocytes) in the brain. Recently published evidence suggests that, in fact, suppression of astrocyte growth may contribute to microcephaly in RS [19]. Thus, the choline PL pattern suggesting reduced growth potential in Mecp2-deficient mouse brain may represent in part non-neuronal cells. Indeed, reduced levels of the astrocyte marker, *myo*-inositol, in the brains of *Mecp2*-/y mice would be consistent with the assumption of a reduced number of astrocytes (see discussion of osmolytes below). While the interpretation of our PtdC data in terms of reduced cell growth is straight forward and rather compelling, the extent to which astrocytes and neurons are responsible for growth reduction awaits further clarification (Table S2). Experiments on co-cultured *Mecp2*-/y astroglial cells and neurons could be performed to study the relationship between metabolism and growth in each cell type [37]. Immunohistochemistry of brain tissue sections from *Mecp2*-/y mice may be employed to test the hypothesis of a more pronounced growth impairment in astrocytes vs. neurons, although correct quantitation of astrocytes may be rather difficult for reasons outlined elsewhere [38].

### Potential role of PAF-remodeling in neurotransmission of Mecp2-deficient mice

PL metabolism is not only essential to cell growth. PL remodeling plays an important role in the generation of lipid messengers and in intracellular signaling. The characteristic differences in choline PL containing diacyl and acyl-alk(en)yl moieties between Mecp2-deficient mice and controls point to modifications of the platelet activating factor (PAF) remodeling pathway or “PAF cycle” (Figure S2) [39]. PAF is an acyl-alkyl PL that modulates a range of cellular processes. PAF effects relevant to the CNS may include enhancement of glutamate exocytosis, neuronal migration, memory formation, and collapse of growth cones essential to the formation of synapses (Table S2) [39]. While our results support the hypothesis of an altered PAF cycle in the *Mecp2*-null mouse brain, we have no direct evidence of changed PAF concentrations since PAF levels are below the detection threshold of our MRS experiments. Highly sensitive techniques such as mass spectrometry should be employed to reveal whether the levels of PAF (and a number of PAF metabolites) are indeed modified as a result of the perturbations detected in this report. This would be of particular interest considering that PAF is known to activate excitatory synapses by increasing presynaptic glutamate release. Future studies have to determine if PAF plays a role in RS.

### Mecp2 deletion causes characteristic changes in glutamate metabolism

Altered glutamine/glutamate ratios found in the *Mecp2*-null mouse brain may be explained based on previously published evidence of heightened excitatory activity in girls with RS [40]. The latter finding indicates that glutamate production and release from neurons are enhanced, while normal postsynaptic signal transduction may be disrupted due to overexcitation by increased glutamate release. Perturbed signaling and/or dysregulation mediated by astrocyte-associated glutamate receptors [41] may trigger increased glutamate uptake and glutamate-glutamine conversion in astrocytes, increasing glutamate-glutamine cycling (Figure S3). This would be consistent with (i) data showing an increase in metabolic activity in glia upon increased glutamate release from neurons [42], and (ii) previously reported reduction of brain ATP levels in *Mecp2*-null mice [20] since ATP is also consumed in the synthesis of glutamine from glutamate [43]. Glutamate levels may be kept constant by compensatory glutamate synthesis from glucose and by anaplerosis (Table S2) [44], [45]. In fact, the glutamate-glutamine cycle and neuronal activity are tightly linked to glucose metabolism (Figure S3), and elevated glutamate concentrations found in the CSF of RS patients have also been interpreted in terms of enhanced glucose utilization [22].

An alternative explanation for increased glutamine levels in *Mecp2*-null mice may be a reduction in glutaminase activity, caused by a decreased need for glutamate neurotransmitter production due to a reduced number of synapses [17]. The latter might be a consequence of reduced dendritic arborization [40]. Also in this model, compensatory glutamate synthesis may maintain stable glutamate levels as suggested above. While a perturbation of the glutamate-glutamine cycle in the *Mecp2*-null mouse brain is well supported by our data, mechanistic details remain to be elucidated. A quantitative investigation of the relationship between glutamate-glutamine cycling, glucose and energy metabolism in RS would have to include flux measurements based on ^13^C-labeled glucose as a precursor, and would also have to consider a possible role of GABA [46].

### Osmoregulation is perturbed in Mecp2-deficient mice

*Myo*-inositol levels were markedly decreased in Mecp2-deficient mice vs. controls, in both in vivo [20] and in vitro MRS measurements. Among all the water-soluble compounds quantitated in this study, the *myo*-inositol decrease and glutamine increase occurred with the highest statistical significance. In addition, the levels of both compounds were negatively correlated for all samples combined (p = 0.05, Spearman-Rank test), and *myo*-inositol was decreased by about the same amount (1.21 µmol/g tissue wet weight) as glutamine was increased (1.16 µmol/g). Since both compounds exert an osmoregulatory function, one may speculate that the increased glutamine accumulation discussed above may have triggered a compensatory decrease in *myo*-inositol. Such an effect has been described for cultured astrocytes [45], and may also occur when intracellular glutamine concentration is increased due to other mechanisms caused by *Mecp2*-deletion. Since *myo*-inositol is considered to be an astrocyte marker, a decreased *myo*-inositol level in Mecp2-deficient mice may alternatively indicate a lower number of astrocytes rather than a compensation for glutamine increase (see above and Table S2). However, a drastic reduction of the number of astrocytes vs. neurons is unlikely because in this case, a significant decrease in creatine would have been observed for Mecp2-deficient mice as creatine is more expressed in astrocytes than in neurons [47]. Further studies are necessary to clarify this point, as suggested above in the discussion of PL metabolism and cell growth. Future studies with alternative *Mecp2* mutations (nonsense/missense, overexpression) should reveal whether the observed *myo*-inositol effect is specific to the particular animal model employed [20]. Reduced *myo*-inositol levels have not been reported in the brains of RS patients to date; however, only a few case reports are available on subjects with variable age, while brain *myo*-inositol concentrations are known to change with age even in healthy children [48].

### Conclusion

The quantitative analysis of a large number of brain metabolites allowed us to obtain, for the first time, a comprehensive characterization of the metabolic phenotype associated with *Mecp2* mutation. Most importantly, metabolic fingerprints of reduced choline phospholipid turnover were detected, implying diminished cell growth in agreement with reduced brain size (see also Table S2). Moreover, indicators of altered osmoregulation in *Mecp2*-null mouse brain suggest another link to changes in brain size, potentially caused by neurons and astrocytes. In addition, changed neurotransmitter metabolism hints at specific mechanisms of brain dysfunction due to *Mecp2* deletion. These hypotheses open new avenues for studying the genotype-phenotype correlation in RS, and for the identification of novel molecular targets for more efficient pharmacological RS treatment. They also serve as a basis for future studies investigating brain metabolism across the age spectrum of the animals, including measurements at a presymptomatic stage with the potential to distinguish between primary and secondary effects of *Mecp2* deletion.

## Materials and Methods

### Animals

Experiments were performed using the mouse model strain B6.129P2(C)-*Mecp2* ^tm1-1Bird^ as described previously [20]. Briefly, the mice obtained from Jackson Laboratory were maintained on a C57BL/6 background. Hemizygous mutant males were generated by crossing heterozygous knockout females to C57BL/6 males. All experiments were performed in hemizygous Mecp2-deficient males of 5–8 weeks of age. Although RS in humans predominantly affects female patients, most researchers use Mecp2-/y male mice for their studies. This choice is dictated by the fact that the *Mecp2* gene is X-linked in mouse and in humans, and females will thus have a different amount of normally Mecp2-expressing cells depending on their X-chromosome inactivation profile. Since the proportion of *Mecp2*-deleted X chromosomes that will be inactivated in a given female animal is unpredictable, we decided to use *Mecp2*-/y male mice that correspond to a complete absence of the *Mecp2* gene product in all cells (i.e., a real null phenotype) [20]. One consequence of complete Mecp2 suppression is that these animals are extremely frail, and therefore are difficult to reproduce in large numbers. Hence, while *Mecp2*-/y males are a widely accepted RS model, it is not an ideal model. Interestingly, a recent publication comparing a male and a female RS model demonstrated that both models exhibited very similar somatic growth and behavioral changes [49]. In this pilot study, four *Mecp2*-/y mice and four C57BL/6 controls were used. The experimental procedures were carried out in keeping with the European Guidelines for the Care and Use of Laboratory Animals (Council Directive 86/6009/EEC).

### Sample preparation

Animals were sacrificed by cervical dislocation. The brain was swiftly and entirely removed through an incision in the skull, freeze-clamped, weighed, and stored at −80°C until extraction with methanol/chloroform/water (4∶4∶4 ml). The frozen brain was broken up into several pieces in a mortar under liquid nitrogen. The brain pieces obtained were consecutively added to 4 ml ice-cold methanol and immediately homogenized using a Polytron tissue homogenizer (Kinematica, Kriens-Lucerne, Switzerland). The sample was incubated on ice for 15 min, then 4 ml of ice-cold CHCl_3_ and 4 ml 4°C water were added sequentially, under vigorous shaking after each addition. Samples were thoroughly vortexed and placed at −20°C overnight for phase separation. Subsequent centrifugation at 11,000 rpm (ca. 13,000 g at max. radius) in a Sorvall Evolution RC centrifuge (Thermo Electron Corporation, Waltham, MA, USA) for 40 min at 4°C completed the phase separation.

Solvents were evaporated from the chloroform/methanol phase under a nitrogen stream, and methanol was removed from the water/methanol phase by bubbling nitrogen through the solution while samples were kept on ice. The aqueous solution was lyophilized overnight, and both lyophilizate and lipid samples were stored at −80°C. For NMR spectroscopy, lipids were dissolved in a ^2^H-chloroform/methanol/water solution as described elsewhere [50]. The lyophilizate was redissolved in D_2_O, and pH was adjusted to *ca.* 7.0. The sample was placed in a 5-mm Wilmad NMR tube (528-PP; Carlo Erba-SDS, Val de Reuil, France) containing a stem coaxial insert (2 mm O.D.) filled with a D_2_O solution (pH 7.7) of 20 mM sodium 3-(trimethylsilyl)-2,2′,3,3′-tetradeuteropropionate (TSP-d_4_) for ^1^H MRS, and 20 mM methylenediphosphonate (MDP) for ^31^P MRS, for chemical-shift referencing and quantitation. Chemicals were purchased from Sigma-Aldrich (Saint Quentin, Fallavier, France), except for a number of phospholipids used for MRS signal assignment (Doosan Serdary Research, Toronto, ON, CA).

### NMR spectroscopy

^1^H and ^31^P NMR spectra at 400.1 and 162.0 MHz, respectively, were obtained on a 9.4 T AVANCE 400 WB FT-NMR spectrometer from Bruker (Wissembourg, France), and analyzed with Bruker's Topspin software (for more technical details see Text S1). Metabolite concentrations are given as µmol/g brain tissue (wet weight). In addition, ratios of metabolite levels are presented where appropriate.

### Statistics

The statistical methods used to detect significant differences and correlations between groups included nonparametric tests (Statview 5.0.1, Cary, NC, USA). Parametric tests such as analysis of variance are appropriate only where the conditions of equal variances and normal distribution are met. However, Statview's F-test procedure yielded unequal variances for several metabolite concentrations and metabolite ratios, and common tests for normal distribution do not give meaningful results when applied to small sample groups [51], [52]. Consequently, we consistently used the nonparametric Mann-Whitney *U* test to determine the significance of differences between two groups, and the nonparametric Spearman-Rank test for correlations between metabolite levels (for further statistical details see Table S1).

## Supporting Information

Text S1 Technical details and parameters for NMR spectrum acquisition and evaluation
(0.03 MB DOC)

Table S1 Comparison of statistical results from parametric vs. nonparametric tests
(0.04 MB DOC)

Table S2 Pathways and potential biochemical/biological effects associated with metabolites measured in brains of *Mecp2*-/y and control mice.
(0.02 MB DOC)

Figure S1 The Kennedy pathway of phosphatidylcholine synthesis and degradation. Levels of metabolites that were significantly increased (decreased) in the brains of Mecp2-deficient mice vs. controls are indicated by upward (downward) arrows (no difference was detected for underlined metabolites). Cells regulate membrane PtdC turnover by coordinating the opposing actions of PtdC synthesis (center) and PtdC degradation (left) [35]. In this way, resting cells (that constitute the majority of adult brain cells) rapidly synthesize and degrade PtdC, maintaining a constant PtdC mass [35]. During this stationary PtdC turnover that requires efficient PtdC degradation, cells tend to sustain relatively high levels of the PtdC degradation product, GPC, with PC/GPC ratios being relatively low [34], [53]. Thus, the combination of high PtdC levels and PC/GPC ratios indicates low PtdC turnover, in agreement with an established model described elsewhere (refs. 32, 33, 50, 51). This link between PL profiles and PL turnover allows the detection of changes in PL turnover in the absence of *direct* turnover rate measurements [34], [35], [53], [54]. High PtdC turnover rates in the *resting* state allow cells to efficiently switch to PtdC accumulation for membranesynthesis when in a *growing* state (e.g. as soon as they reach the S phase), simply by blocking PtdC degradation [35]. Several lines of evidence indicate that this mechanism is crucial to normal cell proliferation [54]. In light of this model, increased PtdC accumulation in the *Mecp2*-null mouse brain, in conjunction with decreased levels of the PtdC degradation product, lyso-PtdC, as well as increased PC/GPC ratios, indicates a reduced ability to degrade PtdC, resulting in restricted PtdC production in cells when in a growing state [35].
(2.25 MB TIF)

Figure S2 The PAF cycle (adapted from [37]). Levels of metabolites that were increased (decreased) in the brains of Mecp2-deficient mice vs. controls are indicated by upward (downward) arrows. The concentration of underlined metabolites did not vary between the two groups. The first step in PAF generation is the formation of lyso-PAF from AAPtdC (I). AAPtdC can then be resynthesized from lyso-PAF, potentially via lyso-PtdC (II). Subsequently, lyso-PAF needs to be regenerated either by phospholipolysis (III), or by forming PtdEplasm from AAPtdC (IV). AAPtdC levels were significantly increased in the brains of Mecp2-deficient mice vs. controls, indicating reduced AAPtdC degradation and/or enhanced AAPtdC resynthesis from lyso-PAF [52]. Of these two mechanisms, a decrease in AAPtdC degradation is more likely, given that also PtdC accumulated due to decreased degradation (see discussion of PL turnover above and Figure S1). The second step in PAF formation is the acetylation of lyso-PAF (V), with an alternative route being de novo synthesis (VI) in analogy to the Kennedy pathway for PtdC (Figure S1). In de novo synthesis (VI), choline is phosphorylated and PC is transferred via CDP-choline to alkyl-acetylglycerol. Finally, the cycle is completed by the degradation of PAF to lyso-PAF (VII). Overall, our results are consistent with an altered PAF remodeling pathway in Mecp2-deficient brain, where AAPtdC accumulates while *de novo* PAF formation may be increased. Further experiments have to reveal whether PAF levels or turnover rates are indeed changed.
(0.07 MB TIF)

Figure S3 The glutamate-glutamine cycle. The excitatory neurotransmitter, glu, is synthesized in neurons from glutamine by way of glutaminase (E.C. 3.5.1.2) activity. After being released from neurons, glutamate is removed by glial Excitatory Amino Acid Transporters (EAAT) that are increased in younger RS girls (thick arrow), and is taken up by astrocytes together with sodium through an energy-dependent pump. Since an increase in intracellular Na^+^ by cotransport with glutamate stimulates Na,K-ATPase, oxygen consumption, and glucose utilization in astrocytes, glutamate release from neurons causes an increase in metabolic activity in the surrounding glia [42]. Elevated glutamine levels (arrow) and glutamine/glutamate ratios in the brains of Mecp2-deficient mice compared to controls, at insignificant glutamate changes (underlined), are potentially due to enhanced conversion of glutamate to glutamine by glutamine synthetase (E.C. 6.3.1.2) in astrocytes, accompanied by increased glutamate synthesis via the citric acid cycle (thick arrows).
(2.25 MB TIF)
