# Supplementary material for: Metabolic Fingerprints of Altered Brain Growth, Osmoregulation and Neurotransmission in a Rett Syndrome Model
Source: PLoS One. 2007 Jan 17;2(1):e157. doi: 10.1371/journal.pone.0000157 (PMC1766343; doi:10.1371/journal.pone.0000157)
Supplement: Text S1 — Technical details and parameters for NMR spectrum acquisition and evaluation (0.03 MB DOC) [file pone.0000157.s002.doc]

Text S1

Technical details and parameters for NMR spectrum acquisition and evaluation

*Acquisition of NMR spectra*

Spectra were acquired using a quattro nucleus probe for 5-mm tubes (QNP tunable to ^1^H, ^31^P, ^13^C and ^19^F NMR resonance frequencies). Samples were spun during NMR measurements at a spin rate of 20 Hz. A standard Bruker Eurotherm BVT-3200 variable temperature unit was employed to maintain a sample temperature of 28°C. ^1^H and ^31^P NMR spectra were acquired under conditions that ensured negligible signal saturation for all metabolites and the reference compounds, as verified by appropriate test experiments. ^1^H NMR spectra of the aqueous extract phase were acquired during AQ = 3.28 s (64k), preceded by water proton presaturation for 6 s (power setting 0.001 W). Further ^1^H acquisition parameters were : sweep width SW = 12 ppm, pulse repetition time TR = 24.283 s, pulse width (90°) PW = 12.1 μs. Generally, 64 transients were acquired, resulting in a total acquisition time of 26 min. ^31^P NMR spectra of the organic extract phase were acquired during AQ = 3.165 s (4k), using inverse-gated WALTZ16 proton decoupling (power setting 19dB for a pulse width of 100 μs). Further ^31^P acquisition parameters were : sweep width SW = 4 ppm, pulse repetition time TR = 18.2s, pulse width (90°) PW = 9.5 μs. Generally, 1800 transients were acquired, resulting in a total acquisition time of 9 h. A broader sweep width (25 ppm) was employed in experiments including the MDP signal.

*Processing and evaluation of NMR spectra*

Lorentzian/Gaussian lineshape transformation was applied to free induction decays before Fourier transform, phase and baseline corrections. The line broadening parameter, LB, varied between -0.2 and ‑0.6 Hz for ^1^H NMR spectra, and between -1 and -3 Hz for ^31^P NMR spectra. The Gaussian parameter, GB, varied between 0.015 and 0.3 for ^1^H NMR spectra, and between 0.05 and 0.21 for ^31^P NMR spectra. Spectra were integrated using Bruker's MDCON deconvolution routine. A 60-100 % Gaussian lineshape was found to optimally fit the metabolite signals, as a function of the GB and LB parameters chosen.

While ^1^H NMR spectra were sufficiently resolved to quantitate individual molecular species, ^31^P NMR spectra yielded signals representing individual phospholipid classes and subclasses. These are characterized by specific molecular sites close to the observed nucleus, i.e. near the phosphate moiety. Thus, phospholipids are distinguishable based on (i) their polar head (phosphate; choline, ethanolamine, serine or inositol phosphate; terminal phosphoglycerol or phosphate-linked glycerol bridge), (ii) their backbone (glycerol, sphingosine), and (iii) their bonds on glycerol C-1 and C-2 (alcohol, ester, ether or vinyl ether). Structural differences occurring at more distant sites such as unsaturation or peroxidation of fatty acid chains, or variations in chain length beyond a few bonds from the backbone, did not result in well separated ^31^P resonances.
