# Supplementary material for: Metabolic Fingerprints of Altered Brain Growth, Osmoregulation and Neurotransmission in a Rett Syndrome Model
Source: PLoS One. 2007 Jan 17;2(1):e157. doi: 10.1371/journal.pone.0000157 (PMC1766343; doi:10.1371/journal.pone.0000157)
Supplement: Table S1 — Comparison of statistical results from parametric vs. nonparametric tests (0.04 MB DOC) [file pone.0000157.s003.doc]

Table S1

*Comparison of statistical results from parametric vs. nonparametric tests*

|  | Cho | PC | | GPC | | PC/GPC | | PC+GPC | | tCho | | glu | | gln | | gln/glu | |
| --- | --- | --- | --- | --- | --- | --- | --- | --- | --- | --- | --- | --- | --- | --- | --- | --- | --- |
| p | 0.052 | 0.895 | | 0.263 | | 0.053 | | 0.462 | | 0.336 | | 0.445 | | 0.046* | | 0.066 | |
|  | Glx | gly | | asp | | GABA | | *myo*-Ins | | tau | | NAA | | NANA | | *scyllo*-Ins | |
| p | 0.472 | 0.729 | | 0.546 | | 0.111 | | 0.032* | | 0.818 | | 0.720 | | 0.084 | | 0.366 | |
|  | lac | ala | | ac | | suc | | Cr | |  | |  | |  | |  | |
| p | 0.694 | 0.567 | | 0.464 | | 0.714 | | 0.903 | |  | |  | |  | |  | |
|  |  | |  | |  | |  | |  | |  | |  | |  | |  |
|  | PtdC | | AAPtdC | | PtdC_plasm_ | | lyso-PtdC | | CL | | PtdE | | PtdE_plasm_ | | AAPtdE | | SM |
| p | 0.016* | | 0.037* | | 0.072 | | 0.019* | | 0.023* | | 0.050 | | 0.406 | | 0.247 | | 0.152 |
|  | PC-L/PE-L | | PtdI | | PtdS | | PtdG | | PtdA | | lyso-SM | | PLx | | PLy | | PL_tot_ |
| p | 0.116 | | 0.104 | | 0.137 | | 0.393 | | 0.103 | | 0.294 | | 0.454 | | 0.946 | | 0.218 |

The exclusive use of nonparametric methods generally results in an underestimation of significance in cases where the assumptions of parametric tests apply fully or almost completely [49]. Since probability distributions cannot be reliably assessed for small sample groups, we also employed the unpaired Student's *t*-test for equal variances as homogeneity of variances was confirmed for most metabolites (the p values obtained from Student's *t*-test are reported in the table above). Comparison with p values from Tables 1 and 2 shows that (i) no major differences were observed for p values from the parametric *t*-test vs. the nonparametric Mann-Whitney *U* test, and (ii) the parametric *t* test yielded a substantially lower p value for one metabolite only (CL).
