# Supplementary material for: Metabolic Fingerprints of Altered Brain Growth, Osmoregulation and Neurotransmission in a Rett Syndrome Model
Source: PLoS One. 2007 Jan 17;2(1):e157. doi: 10.1371/journal.pone.0000157 (PMC1766343; doi:10.1371/journal.pone.0000157)
Supplement: Table S2 — Pathways and potential biochemical/biological effects associated with metabolites measured in brains of Mecp2-/y and control mice. (0.02 MB DOC) [file pone.0000157.s004.doc]

Table S2 Pathways and potential biochemical/biological effects associated with metabolites measured in brains of *Mecp2*-/y and control mice.

| metabolites | pathways | functions | potential effects |
| --- | --- | --- | --- |
| PC/GPC🡩, PtdC🡩, lyso-PtdC🡫, PC, Cho, GPC | PL synthesis and degradation (Kennedy pathway) | membrane synthesis, cell growth (proliferation; cell size and arborization) | reduced brain size, brain atrophy, retarded brain development, impaired brain function |
| AAPtdC🡩, lyso-PtdC🡫, PtdC🡩, PtdE_plasm_, PC, Cho, GPC, PC/GPC🡩 | de novo PAF synthesis and remodeling | modulation of glu release, neuron development | deregulation of excitatory neurotransmission, neurological dysfunction |
| gln🡩, *myo*-Ins🡫 | osmolyte uptake and release | osmoregulation of neurons (gln) and astrocytes (gln, *myo*-Ins) | alteration in brain size; may indicate changes in cell number or cell size; impaired brain function |
| gln🡩, glu, gln/glu🡩, GABA, gly, asp | amino acid neurotransmitter metabolism, glu-gln cycle | excitatory/inhibitory neurotransmission | perturbation of glutamate transmission, disrupted postsynaptic neurotransmission due to overexcitation, neurotoxicity, neurological dysfunction |

Metabolite levels significantly increased (decreased) in the brains of Mecp2-/y vs. control mice are indicated by an upward (downward) arrow. Unchanged metabolite levels are underlined.
