# Supplementary figures and images for: Metabolic Fingerprints of Altered Brain Growth, Osmoregulation and Neurotransmission in a Rett Syndrome Model
Source: PLoS One. 2007 Jan 17;2(1):e157. doi: 10.1371/journal.pone.0000157 (PMC1766343; doi:10.1371/journal.pone.0000157)

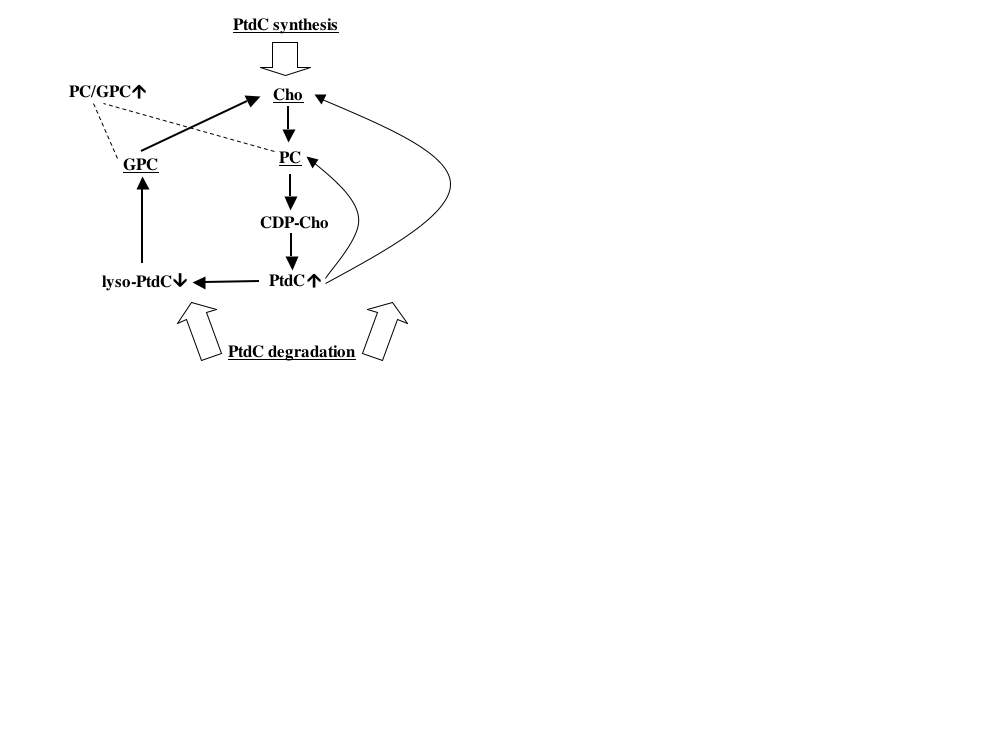

Supplement: Figure S1 — The Kennedy pathway of phosphatidylcholine synthesis and degradation. Levels of metabolites that were significantly increased (decreased) in the brains of Mecp2-deficient mice vs. controls are indicated by upward (downward) arrows (no difference was detected for underlined metabolites). Cells regulate membrane PtdC turnover by coordinating the opposing actions of PtdC synthesis (center) and PtdC degradation (left) [35]. In this way, resting cells (that constitute the majority of adult brain cells) rapidly synthesize and degrade PtdC, maintaining a constant PtdC mass [35]. During this stationary PtdC turnover that requires efficient PtdC degradation, cells tend to sustain relatively high levels of the PtdC degradation product, GPC, with PC/GPC ratios being relatively low [34], [53]. Thus, the combination of high PtdC levels and PC/GPC ratios indicates low PtdC turnover, in agreement with an established model described elsewhere (refs. 32, 33, 50, 51). This link between PL profiles and PL turnover allows the detection of changes in PL turnover in the absence of direct turnover rate measurements [34], [35], [53], [54]. High PtdC turnover rates in the resting state allow cells to efficiently switch to PtdC accumulation for membranesynthesis when in a growing state (e.g. as soon as they reach the S phase), simply by blocking PtdC degradation [35]. Several lines of evidence indicate that this mechanism is crucial to normal cell proliferation [54]. In light of this model, increased PtdC accumulation in the Mecp2-null mouse brain, in conjunction with decreased levels of the PtdC degradation product, lyso-PtdC, as well as increased PC/GPC ratios, indicates a reduced ability to degrade PtdC, resulting in restricted PtdC production in cells when in a growing state [35]. (2.25 MB TIF) [file pone.0000157.s005.tif]

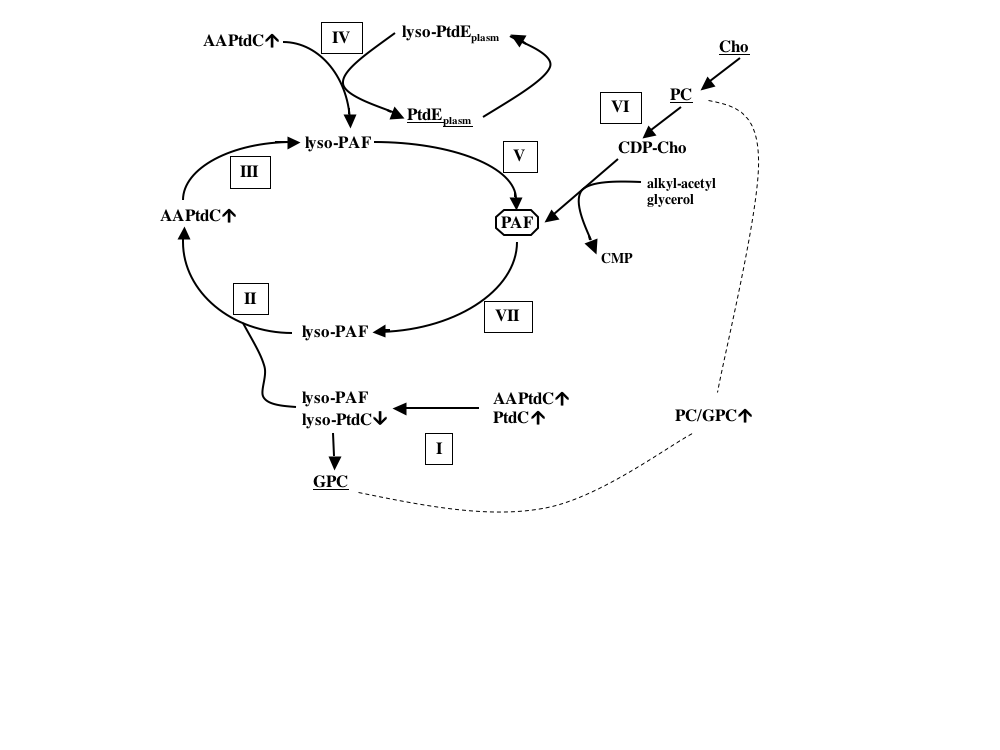

Supplement: Figure S2 — The PAF cycle (adapted from [37]). Levels of metabolites that were increased (decreased) in the brains of Mecp2-deficient mice vs. controls are indicated by upward (downward) arrows. The concentration of underlined metabolites did not vary between the two groups. The first step in PAF generation is the formation of lyso-PAF from AAPtdC (I). AAPtdC can then be resynthesized from lyso-PAF, potentially via lyso-PtdC (II). Subsequently, lyso-PAF needs to be regenerated either by phospholipolysis (III), or by forming PtdEplasm from AAPtdC (IV). AAPtdC levels were significantly increased in the brains of Mecp2-deficient mice vs. controls, indicating reduced AAPtdC degradation and/or enhanced AAPtdC resynthesis from lyso-PAF [52]. Of these two mechanisms, a decrease in AAPtdC degradation is more likely, given that also PtdC accumulated due to decreased degradation (see discussion of PL turnover above and Figure S1). The second step in PAF formation is the acetylation of lyso-PAF (V), with an alternative route being de novo synthesis (VI) in analogy to the Kennedy pathway for PtdC (Figure S1). In de novo synthesis (VI), choline is phosphorylated and PC is transferred via CDP-choline to alkyl-acetylglycerol. Finally, the cycle is completed by the degradation of PAF to lyso-PAF (VII). Overall, our results are consistent with an altered PAF remodeling pathway in Mecp2-deficient brain, where AAPtdC accumulates while de novo PAF formation may be increased. Further experiments have to reveal whether PAF levels or turnover rates are indeed changed. (0.07 MB TIF) [file pone.0000157.s006.tif]

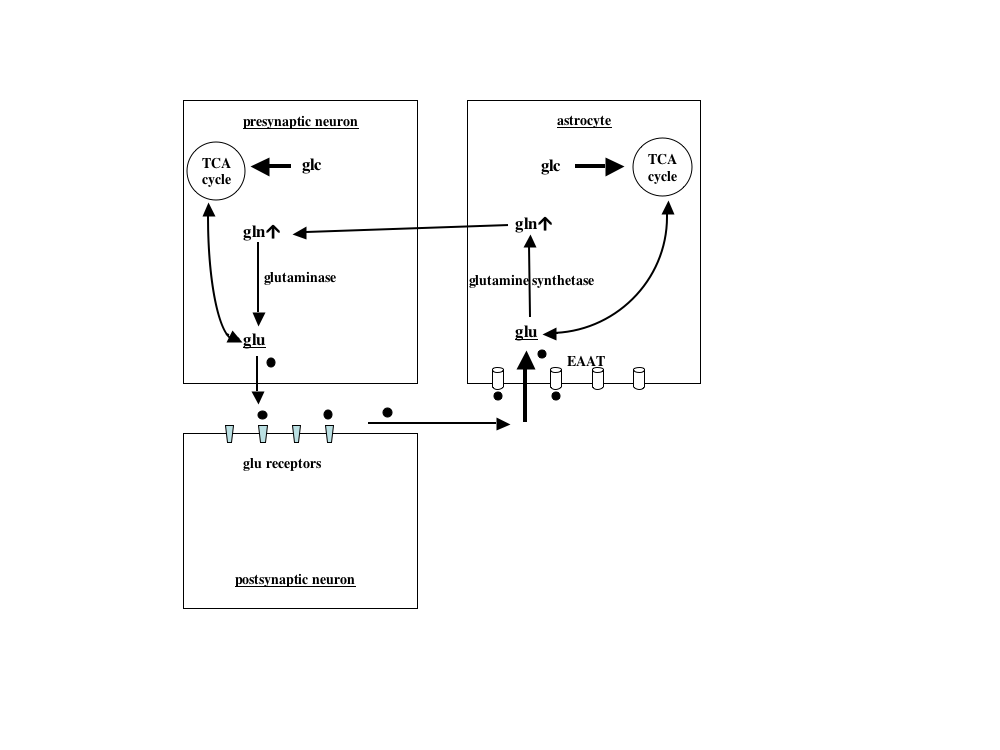

Supplement: Figure S3 — The glutamate-glutamine cycle. The excitatory neurotransmitter, glu, is synthesized in neurons from glutamine by way of glutaminase (E.C. 3.5.1.2) activity. After being released from neurons, glutamate is removed by glial Excitatory Amino Acid Transporters (EAAT) that are increased in younger RS girls (thick arrow), and is taken up by astrocytes together with sodium through an energy-dependent pump. Since an increase in intracellular Na+ by cotransport with glutamate stimulates Na,K-ATPase, oxygen consumption, and glucose utilization in astrocytes, glutamate release from neurons causes an increase in metabolic activity in the surrounding glia [42]. Elevated glutamine levels (arrow) and glutamine/glutamate ratios in the brains of Mecp2-deficient mice compared to controls, at insignificant glutamate changes (underlined), are potentially due to enhanced conversion of glutamate to glutamine by glutamine synthetase (E.C. 6.3.1.2) in astrocytes, accompanied by increased glutamate synthesis via the citric acid cycle (thick arrows). (2.25 MB TIF) [file pone.0000157.s001.tif]
